# Clinical utility of genetic testing in the early diagnosis of Danon disease mimicking hypertrophic cardiomyopathy: a case report

**DOI:** 10.1186/s12872-020-01421-4

**Published:** 2020-04-05

**Authors:** Valeria Novelli, Antonio Bisignani, Gemma Pelargonio, Guido Primiano, Maria Lucia Narducci, Vincenzo Palmieri, Francesco Danilo Tiziano, Paolo Zeppilli, Serenella Servidei, Filippo Crea, Maurizio Genuardi

**Affiliations:** 1 Fondazione Policlinico Universitario A. Gemelli IRCCS, UOC Genetica Medica, Rome, Italy; 2 Istituto di Medicina Genomica, Università del Sacro Cuore, L.go F. Vito 1, 00168 Rome, Italy; 3 Fondazione Policlinico Universitario A. Gemelli IRCCS, Dipartimento di Scienze Cardiovascolari e Toraciche, Rome, Italy; 4 Università Cattolica del Sacro Cuore, Rome, Italy; 5 Fondazione Policlinico Universitario A. Gemelli IRCCS, UOC Neurofisiopatologia, Rome, Italy; 6 Istituto di Neurologia, Università Cattolica del Sacro Cuore, Rome, Italy; 7 Fondazione Policlinico Universitario A. Gemelli, IRCCS, Unità di Medicina dello Sport, Università Cattolica del Sacro Cuore, Rome, Italy

**Keywords:** Danon disease, Genetic testing, Pathogenic variants, Hypertrophic cardiomyopathy, Phenocopy

## Abstract

**Background:**

Danon disease (OMIM 300257) is an X-linked lysosomal storage disorder, characterized by hypertrophic cardiomyopathy (HCM), skeletal myopathy, variable intellectual disability, and other minor clinical features.

This condition accounts for ~ 4% of HCM patients, with a more severe and early onset phenotype in males, causing sudden cardiac death (SCD) in the first three decades of life.

Genetic alterations in the *LAMP2* gene are the main cause of this inherited fatal condition. Up to date, more than 100 different pathogenic variants have been reported in the literature. However, the majority of cases are misdiagnosed as HCM or have a delay in the diagnosis.

**Case presentation:**

Here, we describe a young boy with an early diagnosis of HCM. After 2 episodes of ventricular fibrillation within 2 years, genetic testing identified a novel *LAMP2* pathogenic variant. Subsequently, further clinical evaluations showing muscle weakness and mild intellectual disability confirmed the diagnosis of Danon disease.

**Conclusions:**

This report highlights the role of genetic testing in the rapid diagnosis of Danon disease, underscoring the need to routinely consider the inclusion of *LAMP2* gene in the genetic screening for HCM, since an early diagnosis of Danon disease in patients with a phenotype mimicking HCM is essential to plan appropriate treatment, ie cardiac transplantation.

## Background

Danon disease (DD; OMIM 300257), also named glycogen storage disease type IIb, is an X-linked lysosomal storage disorder, characterized by hypertrophic cardiomyopathy (HCM), skeletal myopathy, variable intellectual disability and retinal disease [1]. This condition accounts for ~ 4% of HCM patients [2], affecting both genders, with a more severe and early onset phenotype in males. Usually, these present HCM at puberty and require heart transplantation around 18–19 years of age, while female carriers develop DCM (dilated cardiomyopathy) or HCM during adulthood often without skeletal muscle involvement [2]. Also, left-ventricular noncompaction phenotype has been reported in a patient affected by Danon disease [3].

Alterations in the *LAMP2* gene, coding for the lysosome-associated membrane protein 2, have been identified as the main cause of Danon disease. So far, more than 100 different pathogenic variants involving the *LAMP2* locus have been described, including non-synonymous variants as well as microdeletions at Xq24 [4]. Here we report on a young male patient, with an initial clinical misdiagnosis of HCM. This case highlights the pivotal role of genetic testing in the rapid diagnosis of this fatal disease.

## Case presentation

A 16-year-old Italian male, with a family history of sudden cardiac death (SCD), was referred to our center with a diagnosis of HCM. His mother died of suspected *postpartum* DCM when she was 24 years old. The proband had received a diagnosis of HCM one year before, when he was admitted for palpitation in a secondary hospital. The ECG showed atrial tachycardia. He underwent two unsuccessful right atrial catheter ablations. Electrophysiological workup was negative for the induction of ventricular arrhythmias. Therefore, he was not considered suitable for ICD implantation at that time.

**Fig. 1:**
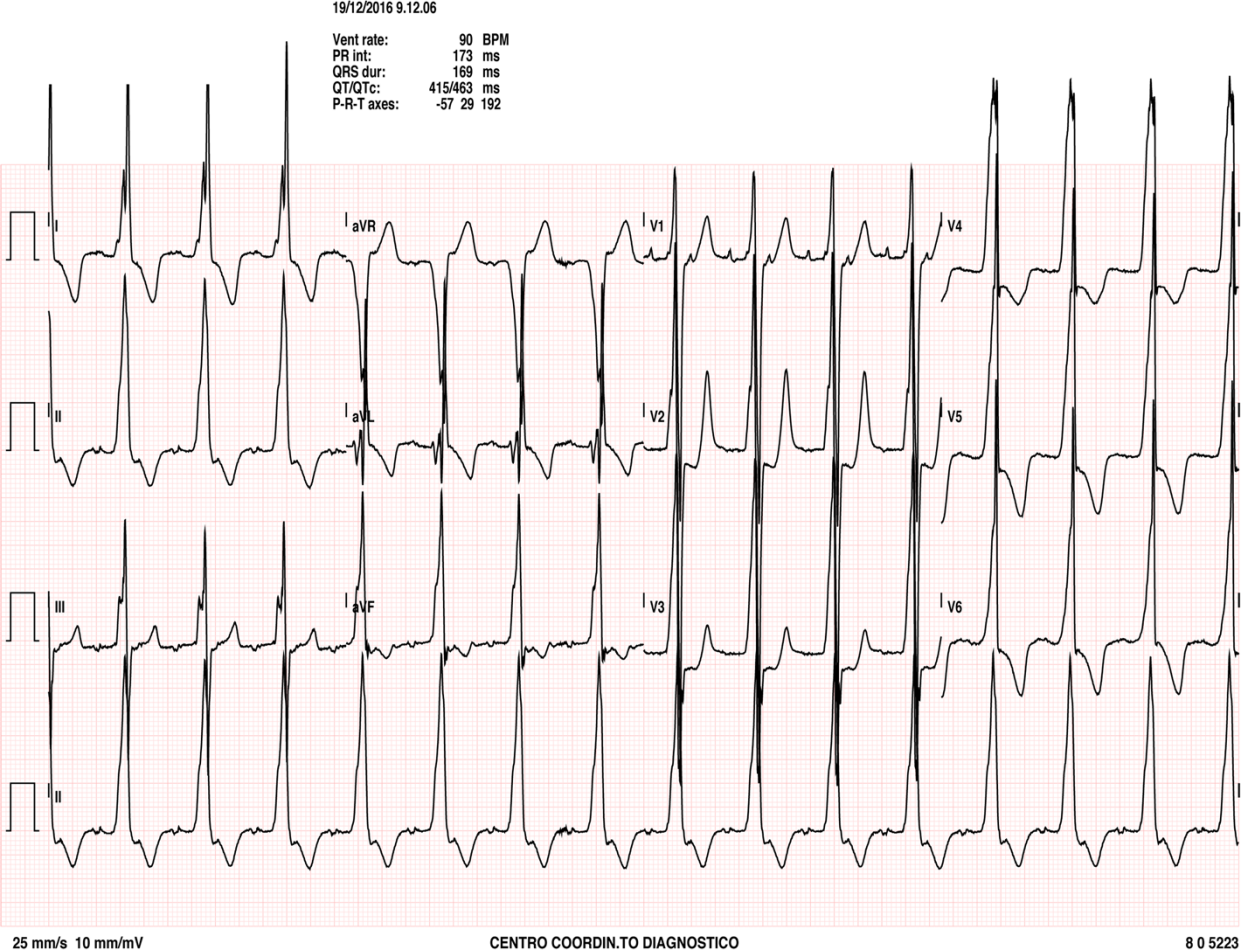
ECG at admission shows atrial tachycardia with 2:1 AV block

**Fig. 2:**
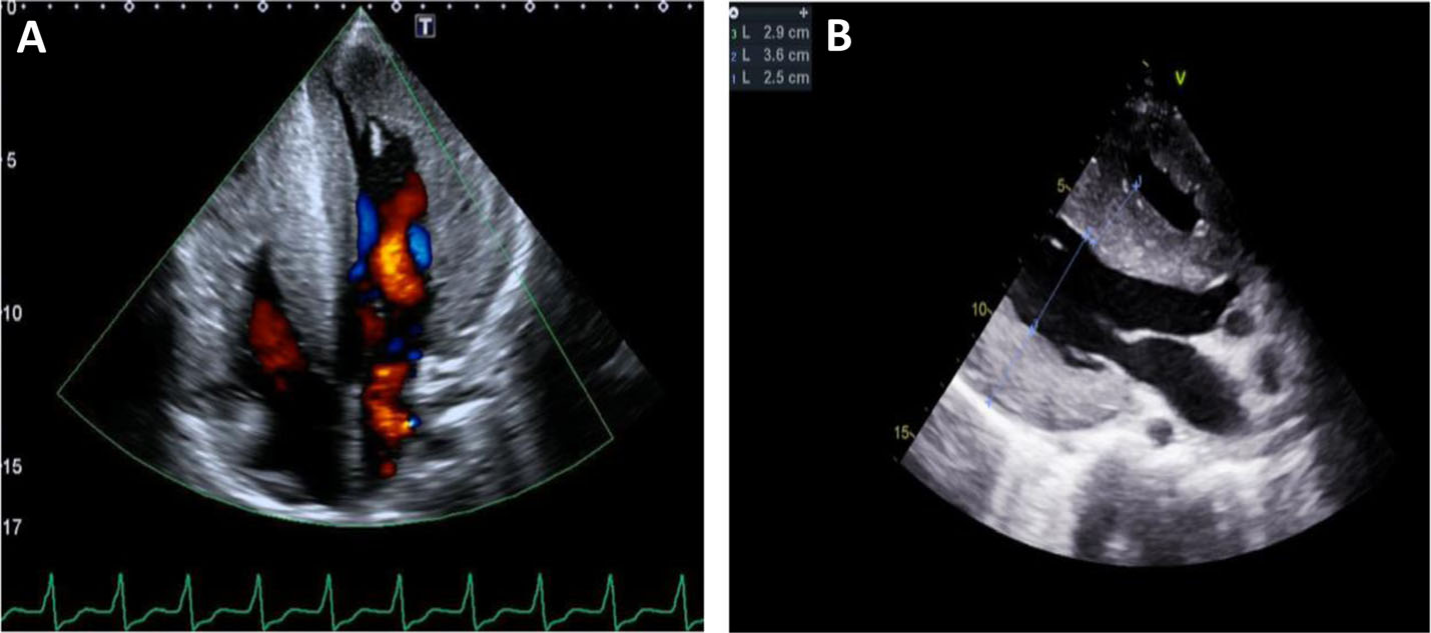
**a-b** Echocardiogram showing apical four chamber (A) and parasternal long-axis (B) views showing severe biventricular hypertrophy with a maximum thickness of 30 mm

Upon admission to our institution, at the age of 17 years, he was symptomatic for palpitations, with evidence of atrial tachycardia (atrial cycle length 330 msec) during ECG monitoring, (NYHA class I) (Fig. 1). The echocardiogram confirmed the severe biventricular hypertrophy (LV septum thickness was 30 mm, PWT 23 mm and LVOT 30 mmHg), with mild LV outflow tract obstruction, previously documented. Moderate biventricular contractile dysfunction was observed, without any evidence of dilation (Fig. 2a-b). Cardiac MRI was performed and confirmed preserved ventricular volumes associated with a severe increase in RV and LV wall thickness and moderate biventricular dysfunction (LVEF 45%; RVEF 42%). The calculated LV mass was 268 g/m^2^. T2w sequences indicated multiple “patchy” areas of increased signal intensity within the biventricular wall, associated with hypoperfusion at rest and late gadolinium enhancement, suggesting multiple foci of fibrosis.

Due to the persistence of symptomatic atrial tachycardia, 3D electroanatomic mapping of atrial tachycardia activation was performed. The earliest atrial activation was recorded close to the area between right pulmonary vein. Subsequently, antral right pulmonary veinelectrical isolation was performed by Smarttouch Navistar catheter (Biosense Webster) (max temp 32 °C, max power 30 W) with interruption of clinical tachycardia. Atrial tachycardia was not re-inducible any longer up to 4 extra-stimuli.

**Fig. 3:**
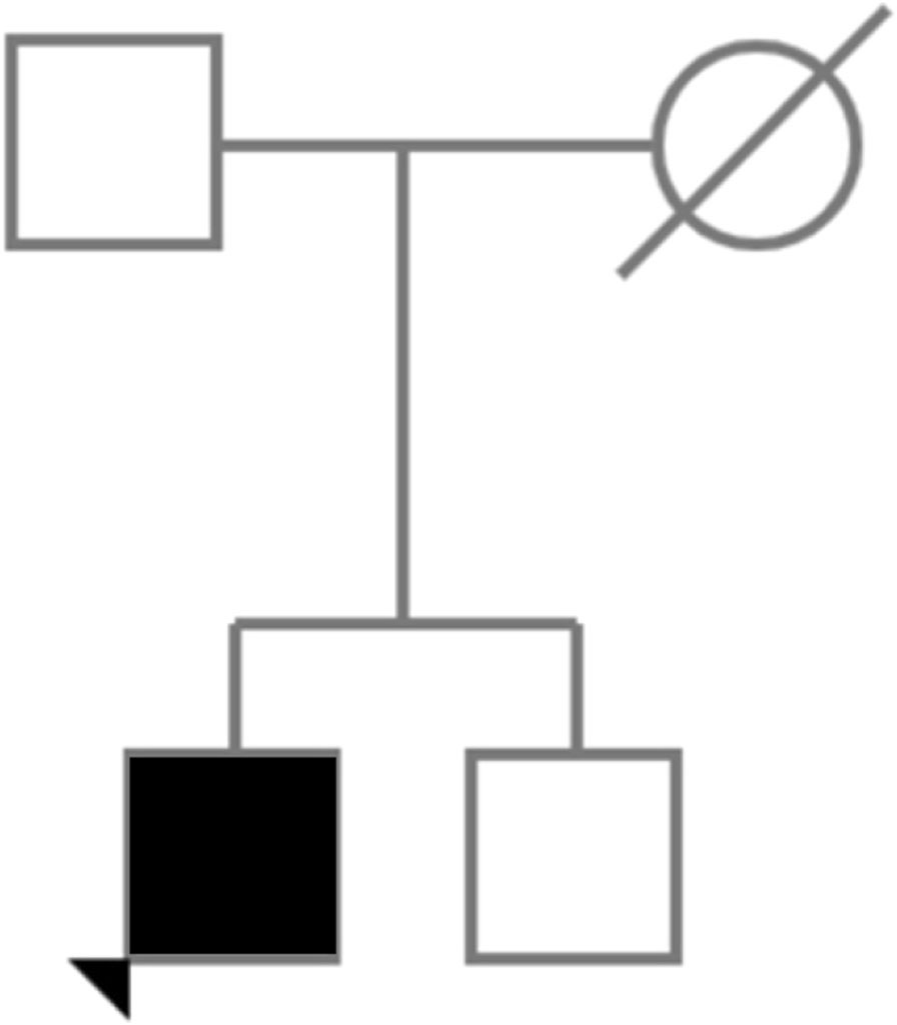
Pedigree of the family

Drug therapy during hospitalization included betablocker (metoprololo 100 mg), diuretic (furosemide12.5 mg) and anticoagulant therapy for 3 months after catheter ablation. Upon admission, genetic counseling was offered to the family and genetic testing was performed (Fig. 3).

Subsequently, further neurological investigations, including skeletal muscle biopsy were performed during this period. These revealed mild intellectual disability, mild weakness of distal upper and lower limb muscles and bilateral *pes cavus*, with an elevated serum CK (562 UI/L). In particular, clinical history revealed intellectual and adaptive functioning deficits in conceptual, social, and practical domains with childhood onset, including difficulties in acquiring academic skills, immature language and the need of assistance in daily living tasks. No other clinical exams was conducted at that time. Finally, since the predicted 5 years risk of SCD was 4.34%, according to the 2014 guidelines on diagnosis and management of HCM, ICD was implanted and muscle biopsy was requested.

### Genetic testing

DNA isolated from peripheral blood leukocytes was screened for genes associated with HCM and related phenotypes using a sequencing panel (Custom Ion Panel, ThermoFisher-US) including 11 genes (*MYBPC3*, *MYH7*, *TNNT2*, *TNNI3*, *TPM1*, *MYL2*, *MYL3*, *ACTC1*, *PLN*, *LAMP2*, *GLA*), encoding for sarcomeric and non sarcomeric proteins. All deep intronic and synonymous coding variants were excluded from further analysis. Then, we filtered out all the variants with an allelic frequency (MAF) of more than 1 × 10^− 4^ in (gnomad.broadinstitute.org). Variant annotation results showed a hemizygous 1 bp deletion, c.453delT, in *LAMP2* (NM_002294), which was confirmed by Sanger sequencing. The variant (chrX:119582932_delT) causes a frameshift in exon 4, p.(F151Lfs*32) resulting in a putative premature stop codon at position 183 of the protein. To the best of our knowledge, it has not previously been reported in the literature, and it is absent in gnomAD and in other electronic databases, including ClinVar (ncbi.nlm.nih.gov/clinvar). Clinical variant interpretation was performed using the ACMG criteria [5]. In particular, the variant was classified as likely pathogenic based on the molecular defect (PVS1; frameshift with premature protein truncation) and on its allele frequency in the general population (PM2).

At that time, genetic counseling was also offered to the older brother of the patient. During counseling, it was explained that probably the mother was a carrier of the LAMP2 pathogenic variant, and that he had a 50% chance of having inherited it. He then agreed to undergo targeted genetic testing for the disease causing variant. The result was negative, indicating that he is not at risk of developing manifestations of Danon disease.

### Muscle biopsy

**Fig. 4:**
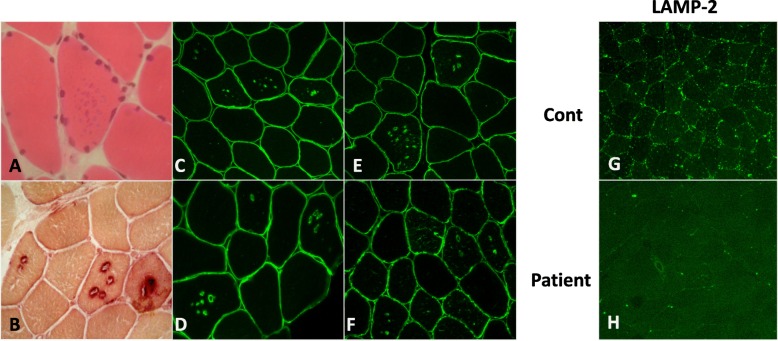
**a-b-c-d-e-f-g-h:** Muscle pathology: hematoxylin and eosin staining **a** shows small basophilic granular structures in the muscle fibers, which are acetylcholine esterase **b** positive autophagic vacuoles; immunohistochemistry using dystrophin **c**, β-sarcoglycan **d**, caveolin-3, and α -dystroglycan **f** antibodies demonstrates that the limiting membranes of the vacuoles have sarcolemmal features; immunohistochemical staining with anti-LAMP2 antibody **g** shows that LAMP-2 expression is present in anunaffected individuaL **h **while is absent in skeletal muscles of the patient

Skeletal muscle biopsy (Fig. 4) demonstrated mild variation in fiber size and numerous scattered small dot-like basophilic granules and vacuoles that showed an enhanced activity of lysosomal acid phosphatase and acetylcholine esterase. The lysosomal vacuoles had the characteristics of autophagic vacuoles with unique sarcolemma features since the limiting membranes were positive for sarcolemma proteins such as dystrophin, sarcoglycans, dystroglycans, and caveolin. Immunohistochemistry using anti-lysosome-associated membrane protein 2 (*LAMP2*) antibodies revealed the absence of the protein in all fibers, concordant with a diagnosis of Danon disease.

## Discussion and conclusions

The case presented herein demonstrates the role of genetic testing in the early diagnosis of Danon disease. A novel *LAMP2* likely pathogenic variant was detected in the proband before a diagnosis of Danon disease was established on clinical grounds.

The natural history of this inherited condition is characterized by a high rate of mortality within the second-third decade of life. Males, in particular, present a rapid progression of HCM with a mean age at death of 19 years (± 6 y). In contrast, females can develop either DCM or HCM with a more protracted course, with a mean age at death of 40 years (± 7 y).

Due to the rapid progression of this lethal disease, early diagnosis is crucial. However, due to the cardiac phenotype that mimics HCM and often obscures the other clinical manifestations, often present in a mild form, early diagnosis can be challenging, in particular in the pediatric population, when the involvement of other organs may not be evident yet. For these reasons, a strategy for a rapid genetic diagnosis is critical to identify patients at risk.

Next generation sequencing analysis using gene panels offers the possibility of investigating simultaneously a large number of genes involved in a genetically heterogeneous condition at affordable costs and with rapid turnaround time. However, the use of larger panels for the systematic screening of HCM offers limited additional sensitivity resulting in an increasing number of variants of uncertain significance (VUS). On the other hand, limiting the analysis to genes associated with non-syndromic HCM, ie sarcomeric genes, might lead to missed or delayed diagnosis in patients affected with syndromic forms of HCM who do not have prominent extracardiac manifestations.

Recently, Alfares et al. [6] analyzing a cohort of 2912 non-syndromic HCM patients, identified 6 cases carrying different *LAMP2* pathogenic variants. These results indicate that Danon disease mimicking an isolated HCM phenotype may be difficult to diagnose on a clinical basis alone.

Furthermore, recent data have shown that inclusion of genes associated with non-syndromic HCM diseases, including *LAMP2*, increases the diagnostic yield of systematic genetic screening for HCM. Analyzing a cohort of 1198 probands with a diagnosis of HCM, it has been observed that *LAMP2* has the same utility of a sarcomeric gene, showing a high frequency of rare pathogenic and likely pathogenic variants in the patient population compared to general population databases, ie ExAC [7].

These data confirm that the inclusion of genes associated with HCM syndromic forms diseases in HCM NGS panels can enable early diagnosis of Danon disease, improves the management of these patients, and allows to identify family members at risk of this fatal condition. Indeed, in this case, after the genetic diagnosis of Danon disease, the patient was immediately placed on a waiting list for heart transplant.

In conclusion, this case underscores the need to routinely analyze the *LAMP2* gene as part of the genetic screening for HCM to assess early diagnosis of Danon disease in patients with a phenotype mimicking isolated HCM. Since in some patients the molecular defect is a large deletion, CNV analysis should also be systematically performed.

## Data Availability

All data generated or analysed during this study are included in this published article.

## Abbreviations

ACMG: American College of Medical Genetics and Genomics
VUS: Variant of uncertain significance
ExAC: Exome aggregation consortium
HCM: Hypertrophic Cardiomypathy
DCM: Dilated cardiomyopathy
CNV: Copy number variation
SCD: Sudden cardiac death
VF: Ventricular fibrillation
ICD: Implanted cardioverter defibrillator
ECG: Electrocardiogram
LV: Left ventricle
MRI: Magnetic resonance imaging
LGE: Late gadolinium enhancementinsert
